# Applying Molecular Modeling to the Design of Innovative, Non-Symmetrical CXCR4 Inhibitors with Potent Anticancer Activity

**DOI:** 10.3390/ijms25179446

**Published:** 2024-08-30

**Authors:** Miquel Martínez-Asensio, Lluís Sàrrias, Gema Gorjón-de-Pablo, Miranda Fernández-Serrano, Judith Camaló-Vila, Albert Gibert, Raimon Puig de la Bellacasa, Jordi Teixidó, Gaël Roué, José I. Borrell, Roger Estrada-Tejedor

**Affiliations:** 1 Grup de Química Farmacèutica, IQS School of Engineering, Universitat Ramon Llull, Via Augusta 390, E-08017 Barcelona, Spain; 2 Lymphoma Translational Group, Josep Carreras Leukaemia Research Institute, E-08916 Badalona, Spain

**Keywords:** CXCR4, virtual screening, molecular design, DLBCL

## Abstract

The identification of new compounds with potential activity against CXC chemokine receptor type 4 (CXCR4) has been broadly studied, implying several chemical families, particularly AMD3100 derivatives. Molecular modeling has played a pivotal role in the identification of new active compounds. But, has its golden age ended? A virtual library of 450,000 tetraamines of general structure **8** was constructed by using five spacers and 300 diamines, which were obtained from the corresponding commercially available cyclic amines. Diversity selection was performed to guide the virtual screening of the former database and to select the most representative set of compounds. Molecular docking on the CXCR4 crystal structure allowed us to rank the selection and identify those candidate molecules with potential antitumor activity against diffuse large B-cell lymphoma (DLBCL). Among them, compound **A**{*17*,*18*} stood out for being a non-symmetrical structure, synthetically feasible, and with promising activity against DLBCL in in vitro experiments. The focused study of symmetrical-related compounds allowed us to identify potential pre-hits (IC50~20 µM), evidencing that molecular design is still relevant in the development of new CXCR4 inhibitor candidates.

## 1. Introduction

The CXC chemokine receptor type 4 (CXCR4) is found in the cells of the hematopoietic system involving multiple functions. CXCR4 is specifically activated by chemokine ligand 12 (CXCL12), also known as stromal cell-derived factor 1 (SDF-1), an 8 kDa chemokine peptide consisting of only 67 amino acids mainly secreted by stromal cells in the bone marrow [1].

The interest in the CXCR4 receptor originally appeared as a potential target to interfere with the human immunodeficiency virus (HIV) life cycle, since CXCR4 acts as a coreceptor during the HIV cell fusion and entry, which starts with the interaction of the virus envelop glycoprotein gp120 with the primary receptor of the host cell, CD4 [2]. However, some years later, CXCR4 was connected with the regulation of hematopoietic stem cell homing to the bone marrow and leukocyte trafficking [3]. The CXCL12/CXCR4 pathway is involved in several aspects of tumor pathogenesis [4], promoting tumor vasculogenesis and angiogenesis in tumor stem-like glioma cells [5] or regulating lymphoid tumor microenvironment in diffuse large B-cell lymphoma (DLBCL) [6].

**Figure 1:**
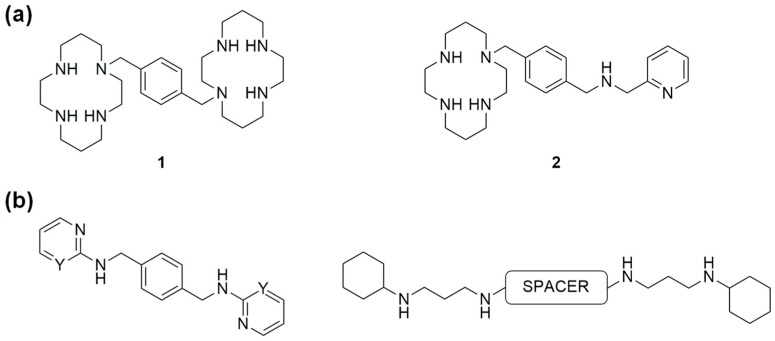
(**a**) Structure of bicyclam AMD3100 (**1**) and monocyclam AMD3465 (**2**); (**b**) example of the general structure of non-cyclam CXCR4 inhibitors reported by Zhan et al. [11] and Fang et al. [12].

Bicyclam AMD3100 (**1**, plerixafor) [7,8] was the first small molecule CXCR4 inhibitor, later commercialized (trade name Mozobil^®^) as an immunostimulant used to mobilize stem cells to the peripheral blood for collection and subsequent autologous stem cell transplantation [9]. Since the discovery of AMD3100, several monocyclam (e.g., AMD3465 [10], **2**) and non-cyclam derivatives have been reported (Figure 1) [11,12].

**Figure 2:**
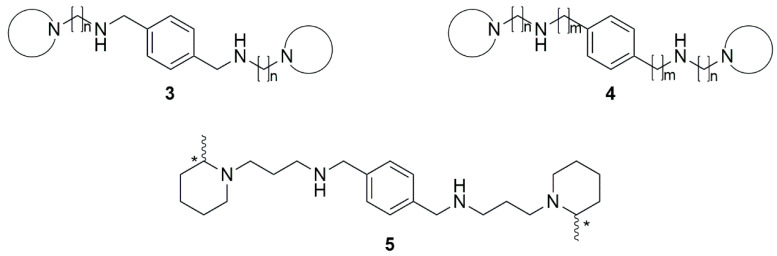
General structure of tetramines **3** and **4** and hit compound **5** (chiral centers are labeled with asterisks).

Our research group has also contributed to the field [13,14,15] by identifying CXCR4 inhibitors with general structure **3** (Figure 2). We have also re-examined if it was necessary to use the *p*-phenylene moiety as the central core to achieve high HIV-1 antiviral activities by synthesizing the more flexible structure **4** [16]. Among these tetraamine derivatives, compound **5** was identified as a hit compound showing promising activity in the in vitro Human CXCR4 Receptor Functional test (IC50 = 0.35 µM), compared to the reference compound **1** (IC50 = 0.26 µM). We synthesized the three stereoisomers of compound **5** (**5**(*R*,*R*), **5**(*S*,*S*), and the meso form **5**(*S*,*R*)) and evaluated their effect on glioma-initiating cells (GICs). The results indicated that compound **5** was effective in preventing tumor initiation and recurrence [17].

Most of our previous works were based on the application of the Structure-Based Drug Design approach by means of a CXCR4 rhodopsin-based homology model built when CXCR4 crystal structures were not available [15]. In 2010, Wu et al. made an important contribution when they published five independent crystallized CXCR4 structures with remarkable resolution (2.5–3.2 Å) [18]. We analyzed in depth the receptor-based virtual screening performance of the five crystallized CXCR4 structures along with our CXCR4 rhodopsin-based homology model, showing that the latter performs comparable to the crystallized structures [19]. By having the structure of CXCR4 available, to the best of our knowledge, the design of new CXCR4 inhibitors seems to have been restricted mainly to the modification of the structure of AMD3100, monocyclams, or other reported non-cyclam small molecule inhibitors with a few exceptions. In these cases, molecular modeling was applied to identify novel potential inhibitors by means of 3D-QSAR [20] or pharmacophore modeling [21]. Otherwise, computational methods have been relegated to ligand–protein docking studies with the aim of elucidating the binding mechanism of empirically found active compounds [12].

**Figure 3:**
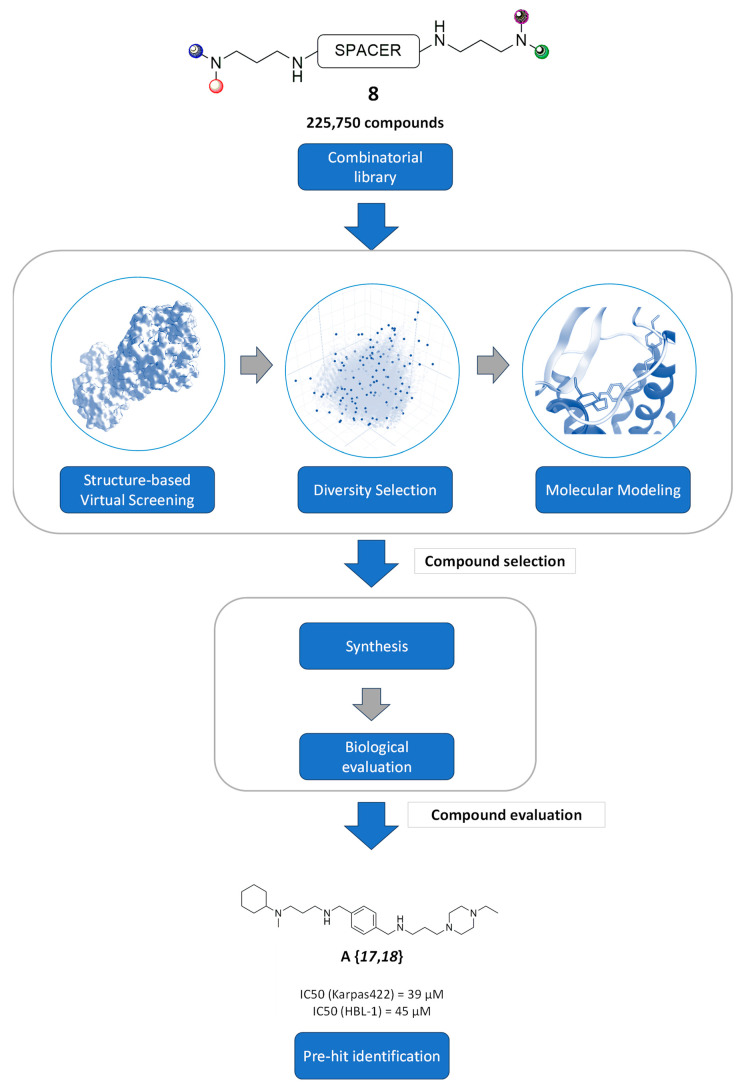
General procedure for the discovery of novel CXCR4 inhibitors. The structure of tetraamines **8** was considered for the creation of the combinatorial library used in the structure-based virtual screening of potential CXCR4 inhibitors. Diversity selection and molecular modeling were used for the identification of those compounds to be synthesized and biologically tested. Finally, compound **A**{*17*,*18*} was identified as a pre-hit.

Therefore, one might ask whether molecular modeling techniques are still useful for the design of new CXCR4 inhibitors. In this study, we challenge the classical virtual screening process by applying it to a combinatorial library constructed by expanding the chemical diversity of tetraamines **3**, **4** considering the generic scaffold **8** (Figure 3). Molecular modeling methods have been applied for the identification of those compounds with a higher probability of becoming potential CXCR4 inhibitors. This procedure has led to the identification of a novel family of compounds with promising activity against DLBCL, demonstrating that virtual screening (based on the application of molecular modeling methods) is still valuable in the early drug discovery stage.

## 2. Results and Discussion

**Figure 4:**
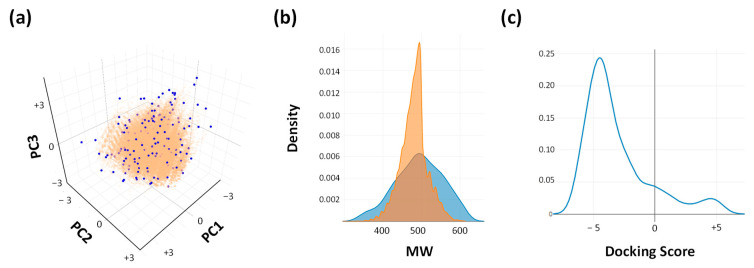
(**a**) Projection of the assessed combinatorial library on the first two principal components. Selected compounds are colored in blue and the space described by the full combinatorial library is in orange. (**b**) Molecular weight distribution for both sets. (**c**) Density distribution of docking scores obtained for the selected molecules.

Given the huge chemical space derived from the combinatorial library, a diversity selection was performed on the molecular descriptor’s space to identify a small molecular subset representative of the whole library. The selection size was set to $\sqrt{N}/2$, which normally offers a good compromise between the space coverage and the number of molecules to handle. The selected 165 molecules are scattered, covering the whole chemical library (Figure 4a) and all of the molecular weight range (Figure 4b).

The binding affinity for selected compounds was predicted by molecular docking, quantified as the score value (Figure 4c). The 10 compounds with better score values were selected as the most promising compounds (molecular structures are available in the Supplementary Materials, Table S2). Interestingly, compounds with the highest score value mainly include spacers **A** and **D**. The length of the central fragment has already been reported to be correlated with the activity of CXCR4 inhibitors [22] and these scaffolds would benefit the interaction with the CXCR4 binding site.

**Figure 5:**
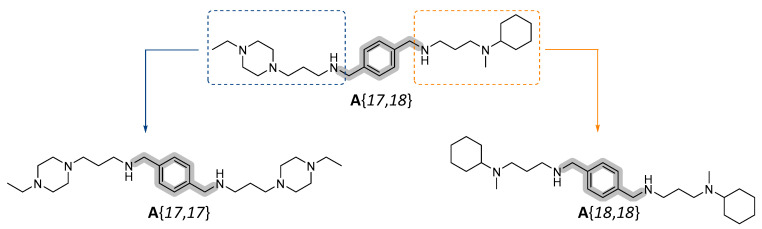
Non-symmetrical compound **A**{*17*,*18*} resulting from the virtual screening protocol and its corresponding two symmetrical compounds **A**{*17*,*17*} and **A**{*18*,*18*}.

A thorough analysis of the synthetic feasibility of selected candidates led us to the identification of compound **A**{*17*,*18*} as the most accessible compound. Note that this compound is a non-symmetrical molecule, composed of the A spacer and amines identified as **9**{*17*} and **9**{*18*} (and consequently referred to as **A***{17*,*18*}). The symmetrical compounds would correspond to **A**{*17*,*17*} and **A**{*18*,*18*} (Figure 5).

### 2.1. Prediction of the Binding Mechanism

**Figure 6:**
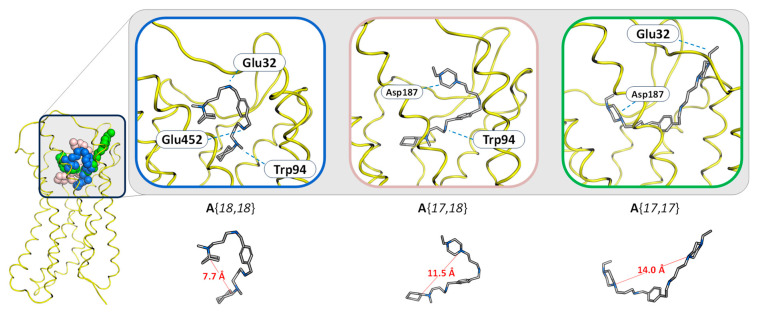
Interaction mechanism predicted by molecular docking and molecular dynamics simulations for compounds **A**{*18*,*18*}, **A**{*17*,*18*}, and **A**{*17*,*17*}.

Induced fit docking followed by molecular dynamics simulation was applied to the selected candidates to evaluate the preferred interaction mechanism with the receptor, considering the molecular flexibility (Figure S2). According to the computational results, although all compounds would mainly interact with the CXCR4 orthosteric binding site (Figure 6), **A**{*18*,*18*} would show slightly higher affinity for CXCR4 (S = −7.9 kcal/mol, LE = −0.25) than **A**{*17*,*18*} (S = −7.5 kcal/mol, LE = −0.23) or **A**{*17*,*17*} (S = −6.8 kcal/mol, LE = −0.21). Interestingly, the presence of an *N*-methylcyclohexanamine fragment (amine **9**{*18*}) would promote a better hydrophobic contact with CXCR4, leading to a curved conformation in which both terminal fragments interact with the inner side of the binding pocket. The intramolecular distance between the two terminal cycles increases progressively when changing the terminal amine **9**{*18*} to **9**{*17*}, showing a decrease in the ability to interact with the internal part of the site (Figure 6). Our results suggest that **9**{*18*} could interact in the CXCR4 subpocket close to the TM2 helix, abutting Trp 94, and extend the interaction to a second subpocket. Depending on the amine on the opposite side of the molecule, the ligand would tend to interact with the TM7 region (Glu 452) in the case of **A**{*18*,*18*} or towards the outside region (Asp187) near TM4 and TM5 in the case of **A**{*17*,*18*}.

Consequently, we decided to synthesize compound **A**{*17*,*18*} and, for comparison purposes, the two symmetrical analogs **A**{*18*,*18*} and **A**{*17*,*17*}.

### 2.2. Synthesis of the Selected Compounds

**Scheme 1:**
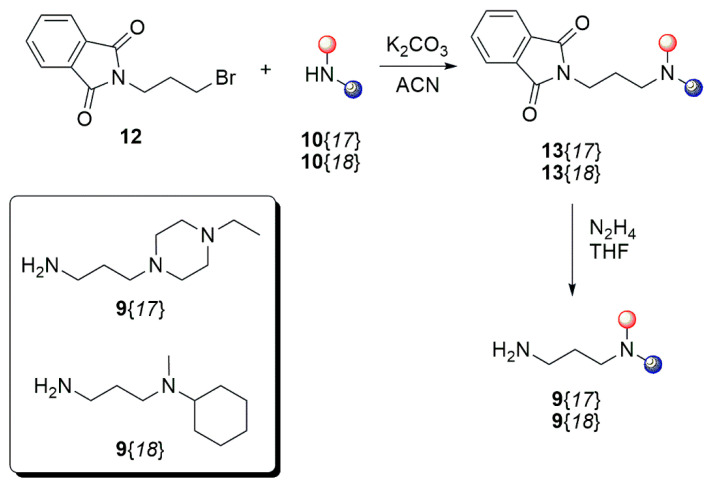
Synthesis of diamines **9**{*17*} and **9**{*18*} from *N*-ethylpiperazine **10**{*17*} and *N*-methylcyclohexanamine **10**{*18*}.

First, we synthesized diamines **9**{*17*} and **9**{*18*} from the corresponding cyclic amines *N*-ethylpiperazine **10**{*17*} and *N*-methylcyclohexanamine **10**{*18*} (Scheme 1). Thus, amines **10**{*17*} and **10**{*18*} were treated with *N*-(3-bromopropyl)phthalimide (**12**) in the presence of K_2_CO_3_ as the base in acetonitrile to afford the corresponding phtalimides **13**{*17*} and **13**{*18*} in 85% and 75% yield, respectively. These later compounds were transformed into the desired diamines **9**{*17*} and **9**{*18*} upon treatment with hydrazine in EtOH in 85% and 42% yield, respectively.

**Scheme 2:**
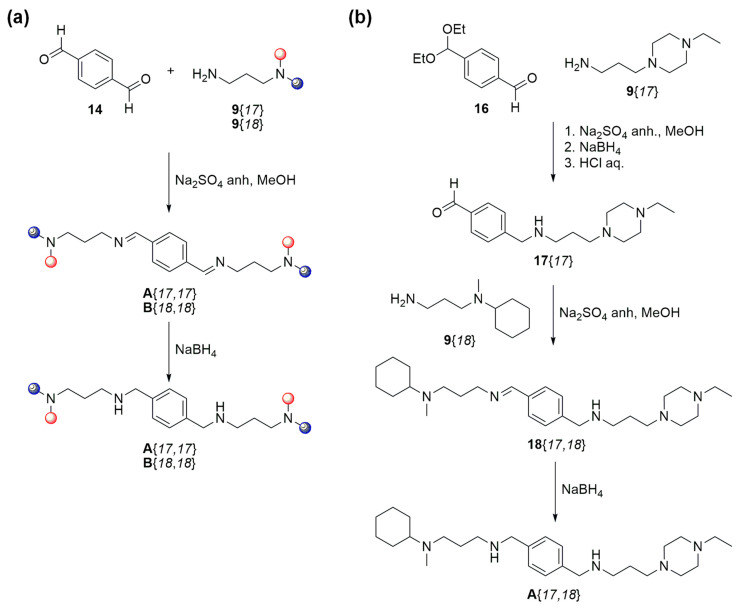
(**a**) Synthesis of symmetrical tetraamines **A**{*17*,*17*} and **A**{*18*,*18*}. (**b**) Synthesis of tetraamine **A**{*17*,*18*}.

On the one hand, the symmetrical tetraamines **A**{*17*,*17*} and **A**{*18*,*18*} were synthesized (Scheme 2a) by using the same protocol described for the synthesis of **5** [13], in which terephtaldehyde (**14**) was treated with two equivalents of the corresponding diamines **9**{*17*} and **9**{*18*} in EtOH at 120 °C and Na_2_SO_4_ as the dehydrating agent to afford the corresponding diimines **15**{*17*,*17*} and **15**{*18*,*18*}, which are directly converted to the desired tetraamines **A**{*17*,*17*} and **A**{*18*,*18*} upon treatment with NaBH_4_ in 51% and 85% yield, respectively.

On the other hand, the non-symmetrical tetramine **A**{*17*,*18*} was obtained starting from 4-(diethoxymethyl)benzaldehyde (**16**) using our previously described protocol for non-symmetrical tetraamines (Scheme 2b) [13]. Thus, **16** was treated with diamine **9**{*17*} in MeOH and Na_2_SO_4_ and the resulting intermediate imine was reduced with NaBH_4_ to the corresponding amine, which was deprotected using 2 M aqueous HCl to afford aldehyde **17**{*17*} in 65% yield. Moreover, **17**{*17*} was converted to tetraamine **A**{*17*,*18*} upon treatment with diamine **9**{*18*} in a second reductive amination, via imine **18**{*17*,*18*}, in 88% yield.

### 2.3. Assessment of the Biological Activity of Selected Candidates

Once the symmetrical compounds **A**{*17*,*17*} and **A**{*18*,*18*} and the non-symmetrical compound **A**{*17*,*18*} were synthesized, their antitumor activity and selectivity were assessed using the MTT assay in a DLBCL cell line of the activated B-cell (ABC) subtype (HBL-1) and in a second cell line of the germinal center B-cell (GBC) subtype (Karpas-422). In parallel, selectivity toward tumor B-cells was evaluated by culturing primary human peripheral blood mononuclear cells (PBMCs) purified from three healthy donors in the same conditions.

**Figure 7:**
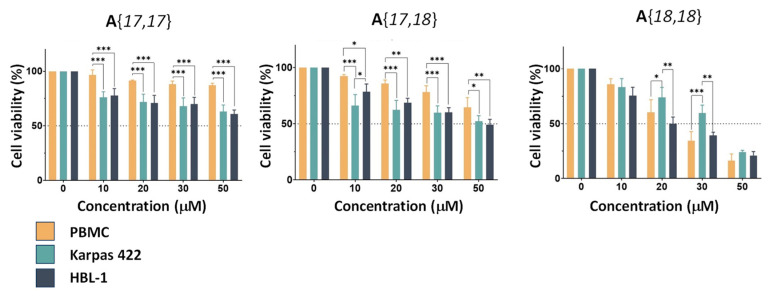
MTT cell viability and proliferation assay results in PBMCs from different healthy donors (*n* = 3) and in Karpas 422 (GCB-DLBCL) and HBL-1 (ABC-DLBCL) cell lines (*n* = 3) at 24 h for all compounds under study. Data are presented as means ± SD. Statistics: One-Way ANOVA statistical test, * *p* < 0.05, ** *p* < 0.01, and *** *p* < 0.001.

As can be seen in Table 1 and Figure 7, the biological activities obtained for the non-symmetrical compound **A**{*17*,*18*} and its corresponding symmetrical derivatives were in agreement with the predictions obtained by molecular modeling: **A**{*18*,*18*} was positioned as the most active (with lowest IC50 values) against Karpas-422 and HBL-1, followed by **A**{*17*,*18*} and **A**{*17*,*17*}. Unfortunately, according to LD50 calculations, **A**{*18*,*18*} compound showed significant toxicity when tested against healthy PBMCs, therefore illustrating poor selectivity. It is noteworthy that, although **A**{*17*,*18*} showed a more moderate activity than **A**{*18*,*18*}, it exerted lower toxicity in normal cells, therefore offering a greater therapeutic window.

Interestingly, **A**{*17*,*18*} and **A**{*18*,*18*} represent the first examples of CXCR4 inhibitors with exocyclic nitrogen synthesized and evaluated in our research group. However, in the literature, there are some examples of symmetrical compounds with exocyclic nitrogen with remarkable activity against CXCR4 (Figure 1b), e.g., WZ811 [23] or HF50731 (IC50 = 19.8 nM) [12]. It is worth noting the high similarity between HF50731 and **A**{*18*,*18*}, especially because the latter was identified, independently, from the analysis of a diversity selection, being the originally identified non-symmetrical compound. Unlike the studies of specific chemical libraries, the computational approach followed in this study offers the possibility to expand the chemical space explored, including non-symmetrical compounds.

## 3. Materials and Methods

### 3.1. Combinatorial Library Enumeration

**Figure 8:**
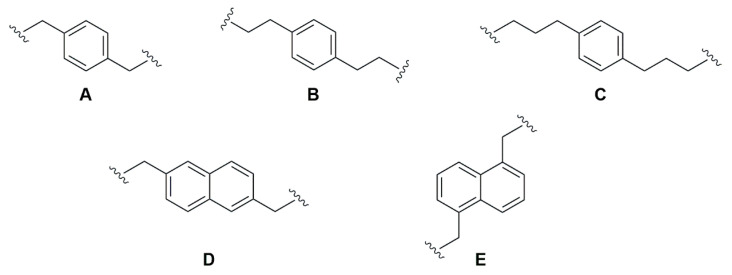
Molecular structure of spacers (**A**–**E**) chosen for the creation of the virtual combinatorial library.

Attending the general structure **8**, a total of five symmetrical spacers (**A**–**E**) was considered, including dialkyl disubstituted phenyl or naphthyl rings, taking into account the previous experience of our research group [13,14,15] (Figure 8).

For the construction of the final chemical library, 300 commercially available cyclic diamines **9** were retrieved from eMolecules (https://www.emolecules.com/, accessed on 26 June 2023) and curated based on their functional groups to guarantee the formation of **8**. The virtual library was enumerated using the Combinatorial Library tool available in Molecular Operating Environment (MOE, 2022.02 Chemical Computing Group ULC, 910-1010 Sherbrooke St. W., Montreal) software. This tool allows the combination of different molecular libraries according to the specified connection information, determining where the functional groups have to be attached to each scaffold. The combination of the spacers and amines led to a combinatorial library initially composed of 450,000 (300 × 5 × 300) derivatives and reduced to 225,750 non-redundant structures due to symmetry, including symmetrical and non-symmetrical compounds.

Lipinski’s rule of five was considered to select only those compounds with drug-like properties, leading to a virtual library of N = 109,044 compounds.

The obtained virtual library was therefore described with 204 2D molecular descriptors, including structural, topological, and physicochemical descriptors (see the Supplementary Materials, Table S1). To gain a better understanding of the information, we performed a principal component analysis (PCA) to reduce the chemical space dimensionality to 7 principal components (accounting for 70% of the data variance). Finally, the size of the virtual library was also reduced to a more manageable number of molecules by conducting a diversity selection of the most dissimilar 165 ($\sqrt{N}/2$) compounds. Library selection was carried out using a distance-based cluster analysis implemented in MOE2022.02 software, defining the Euclidean distance as a metric. Considering the intramolecular distance as a definition of molecular similarity, this tool allows us to rank and select the most dissimilar molecules such as those with the largest minimum intramolecular distance. In our study, intramolecular distance was calculated using the Euclidean distance in the principal components space.

### 3.2. Molecular Docking

The binding mechanism of selected compounds was assessed by molecular docking considering the monomeric CXCR4 (PDBID: 3ODU [18]), removing the crystallization waters and chain B. Hydrogen atoms were added to the receptor with a protonate 3D tool. All the procedures were performed using MOE.

For the virtual screening campaign, the triangle matcher placement method was applied to generate 1000 interacting poses, and their affinity was assessed by the London dG scoring function. Scoring functions attempt to estimate the free energy of binding of the ligand from a given conformation. The best poses were retained and ligand efficiency (LE) was calculated as the quotient between the docking score and the number of heavy atoms in the molecule.

In the case of the selected candidate, the protocol described above was complemented with a refinement stage in which the best 100 poses were refined using the GBVI/WSA dG scoring function and under induced fit conditions. In contrast to the previous scoring function, GBVI/WSA dG is a forcefield-based scoring function that has been demonstrated to be very useful in predicting the binding mechanism of ligands [24].

The co-crystallized IT1t inhibitor (ITD), reported in the PDB, was taken as a reference for the validation of the docking protocol. Docking conditions were also applied on IT1t to corroborate that the docking methodology was able to reproduce the binding mechanism of reported data and was useful. The validation docking led to an RMSD of 0.96 Å and LE = −0.33 (Figure S1 in the Supplementary Materials), demonstrating that the protocol would be useful in predicting the binding mechanism of potentially selected inhibitors.

### 3.3. Molecular Dynamics Simulations

The dynamic behavior of the interacting conformations resulting from molecular docking was assessed by molecular dynamic (MD) simulations for the receptor–ligand complex. All MD simulations were carried out with AMBER 20 software [25] running on the NVIDIA RTX3060 GPU, defining a time step of 2 fs. AM1-BCC atomic charges were calculated for ligand molecules using an antechamber module in AMBER 20 [25]. The molecular system was neutralized and prepared using the tleap program [25] using the AMBER ff14 forcefield for the protein and the GAFF forcefield for ligand molecules. An explicit solvent model was considered by defining a truncated octahedral periodic box of TIP3P water molecules (defining a cutoff of 10 Å). Water molecules were energy minimized before minimizing the energy of the whole system during 1500 cycles using the steepest descent. The system was heated to 300 K at constant volume for 100 ps without restraints. Langevin dynamics with a collision frequency of 1 ps^−1^ was used. The density stage at constant pressure at 1 atm (NVP) was conducted for 100 ps before applying an equilibrium stage of 1 ns at constant temperature (NVT). Finally, 200 ns of production was carried out at a constant temperature. Chemical bonds involving hydrogen atoms were constrained with the SHAKE algorithm [26]. Particle Mesh Ewald (PME) [27] was used in all calculations with a 9 Å long-range cutoff.

### 3.4. General

All solvents and chemicals were reagent grade. Unless otherwise mentioned, all solvents and chemicals were purchased from commercial vendors and used without purification: Fluka (Honeywell Specialty Chemicals, Seelze, Germany), Sigma-Aldrich (Merck Life Science, Madrid, Spain), ABCR (Karlsruhe, Germany), and ACROS Organics (Thermo Fisher Scientific, Madrid, Spain) ^1^H and ^13^C-NMR spectra were recorded on a Varian 400-MR spectrometer (Varian, Palo Alto, CA, USA) (^1^H-NMR at 400 MHz and ^13^C-NMR at 100.5 MHz). Chemical shifts were reported in parts per million (δ) and are referenced to tetramethylsilane (TMS) in ^1^H-NMR spectra and to the residual signal of the solvent CDCl_3_ (77.0) in ^13^C-NMR spectra. Coupling constants are reported in Hertz (Hz). Standard and peak multiplicities are designed as follows: s, singlet; d, doublet; dd, doublet of doublets; dt, doublet of triplets; t, triplet; q, quadruplet; quint, quintuplet; and br, broad signal. IR spectra were recorded in a Nicolet Magna 560 FTIR spectrophotometer and a Thermo Scientific Nicolet iS10 FTIR spectrophotometer (Thermo Fisher Scientific, Waltham, MA, USA) with Smart iTr. Wavenumbers (ν) are expressed in cm^−1^. MS data (*m*/*z* (%), EI, 70 eV) were obtained by using an Agilent Technologies 5975 spectrometer (Agilent, Santa Clara, CA, USA) and a Hewlett Packard HP5988A quadrupole mass spectrometer (Palo Alto, CA, USA) operating in electronic ionization (EI) mode at 70 eV and 4 kV accelerating potential, on a VG AutoSpec (Micromass Instruments, Manchester, UK) Triosector EBE spectrometer operating in Fast Atom Bombardment (FAB) mode. HRMS data were obtained by using a VG AutoSpec (Micromass Instruments) Trisector EBE high-resolution spectrometer (EI or ESI-TOF mode). Elemental microanalyses were obtained on a Carlo-Erba CHNS-O/EA 1180 (Thermo Fisher Scientific) and a EuroVector Instruments Euro EA elemental analyzer (EuroVector, Pavia, Italy). Microwave irradiation experiments were carried out in an InitiatorTM (Biotage, Uppsala, Sweden) microwave apparatus, operating at a frequency of 2.45 GHz with continuous irradiation power from 0 to 400 W. Reactions were carried out in 0.5, 2.5, 5, and 20 mL glass tubes, sealed with aluminum/Teflon crimp tops, which can be exposed up to 250 °C and 20 bar internal pressure. Temperature was measured with an IR sensor on the outer surface of the process vial. After the irradiation period, the reaction vessel was cooled rapidly to 50 °C by air jet cooling. NMR and IR spectra of final products **A**{*17*,*17*}, **A**{*17*,*18*}, and **A**{*18*,*18*} are available in the Supplementary Materials (Appendix S1).

### 3.5. Synthesis

**2-(3-(4-ethylpiperazin-1-yl)propyl)isoindoline-1,3-dione 13**{*17*}. A solution of 1-ethylpiperazine (**10**{*17*}) (0.17 mL, 1.33 mmol), *N*-(4-bromoethyl)phthalimide (**12**) (0.3521 g, 1.31 mmol), and anhydrous K_2_CO_3_ (1 g, 7.24 mmol) in anhydrous acetonitrile (20 mL) was heated at reflux for 15fh. Then, the mixture was cooled down to room temperature, filtered, and concentrated in vacuo. A little portion of water was added and the solution was extracted with dichloromethane. The combined organic layers were dried with anhydrous MgSO_4_ and the solvent was removed under reduced pressure to obtain 0.3751 g of 2-(3-(4-ethylpiperazin-1-yl)propyl)isoindoline-1,3-dione (**13**{*17*}) (1.24 mmol, 95%) as a yellow oil. ^1^H-NMR (400 MHz, CDCl_3_) δ (ppm): 7.88–7.79 (m, 2H), 7.76–7.65 (m, 2H), 3.76 (t, J = 6.9 Hz, 2H), 2.64–2.27 (m, 12H), 1.86 (p, J = 6.9 Hz, 2H), and 1.06 (t, J = 7.2 Hz, 3H). ^13^C-NMR (100.5 MHz, CDCl_3_) δ (ppm): 168.6, 133.9, 132.4, 123.2, 56.1, 53.1, 52.7, 52.3, 36.8, 25.3, and 11.9.

**2-(3-(cyclohexyl(methyl)amino)propyl)isoindoline-1,3-dione 13**{*18*}. The procedure was the same as that stated above for **13**{*17*} but was carried out by using 0.6838 g (3.53 mmol) of *N*-(4-bromoethyl)phthalimide (**12**), 0.46 mL (3.53 mmol) of *N*-methylcyclohexanamine (**10**{*18*}), and 1.1 g (7.96 mmol) of anhydrous K_2_CO_3_ in 10 mL of anhydrous acetonitrile to give 0.6035 g of 2-(3-(cyclohexyl(methyl)amino)propyl)isoindoline-1,3-dione (**13**{*18*})(2 mmol, 79%) as a yellow oil. ^1^H-NMR (400 MHz, CDCl_3_) δ (ppm): 7.89–7.79 (m, 2H), 7.70 (m, 2H), 3.72 (t, J = 6.9 Hz, 2H), 2.54 (t, J = 7.2 Hz, 2H), 2.42–2.31 (m, 1H), 2.24 (s, 3H), 1.93–1.55 (m, 7H), and 1.28–0.98 (m, 5H). ^13^C-NMR (100.5 MHz, CDCl_3_) δ (ppm): 168.5, 133.9, 132.3, 123.2, 62.7, 51.3, 37.4, 36.6, 28.5, 26.9, 26.4, and 26.1.

**3-(4-ethylpiperazin-1-yl)propan-1-amine 9**{*17*}. 2-(3-(4-ethylpiperazin-1-yl)propyl)isoindoline-1,3-dione (**13**{*17*}) (0.9684 g, 3.21 mmol) was dissolved in 20 mL of tetrahydrofuran. The solution was treated with 10 mL (206.15 mmol) of hydrazine monohydrate and the mixture was heated at reflux for 18 h. A white solid corresponding to the phthalhydrazide was filtered and the solution was concentrated. After a second filtration, the solvent was removed under reduced pressure to afford 0.2768 g of 3-(4-ethylpiperazin-1-yl)propan-1-amine (**9**{*17*}) (1.61 mmol, 50%) as a yellow oil. ^1^H-NMR (400 MHz, CDCl_3_) δ (ppm): 2.80 (t, J = 6.7 Hz, 2H), 2.63–2.36 (m, 12H), 2.09 (s, 2H), 1.68 (p, J = 6.7 Hz, 2H), and 1.08 (t, J = 7.2 Hz, 3H). ^13^C-NMR (100.5 MHz, CDCl_3_) δ (ppm): 56.9, 53.4, 52.9, 52.4, 41.1, 29.5, and 12.1.

***N*^1^-cyclohexyl-*N*^1^-methylpropane-1,3-diamine 9**{*18*}. The procedure was the same as that stated above for **9**{*17*} but was carried out by using 0.5588 g (1.86 mmol) of 2-(3-(cyclohexyl(methyl)amino)propyl)isoindoline-1,3-dione (**13**{*18*}), 2.7 mL (55.66 mmol) of hydrazine monohydrate, and 13 mL of tetrahydrofuran to give 0.2665 g of *N*^1^-cyclohexyl-*N*^1^-methylpropane-1,3-diamine (**9**{*18*}) (1.56 mmol, 84%) as a yellow oil. ^1^H-NMR (400 MHz, CDCl_3_) δ (ppm): 2.74 (t, 2H), 2.49 (t, 2H), 2.40–2.31 (m, 1H), 2.24 (s, 3H), 1.84–1.74 (m, 4H), 1.69 (s, 2H), 1.65–1.55 (m, 2H), 1.28–1.15 (m, 4H), and 1.14–1.01 (m, 2H). ^13^C-NMR (100.5 MHz, CDCl_3_) δ (ppm): 62.7, 51.4, 40.8, 37.7, 31.4, 28.4, 26.3, and 26.0.

***N*,*N′*-(1,4-phenylenebis(methylene))bis(3-(4-ethylpiperazin-1-yl)propan-1-amine) A**{*17*,*17*}. An amount of 0.0720 g (0.42 mmol) of 3-(4-ethylpiperazin-1-yl)propan-1-amine (**9**{*17*}), 0.0282 g (0.21 mmol) of terephthalaldehyde (**14**), and anhydrous Na_2_SO_4_ were suspended in 5 mL of anhydrous methanol. The mixture was subjected to microwave irradiation for 2 h at 100 °C. The mixture was filtered and 0.0161 g (0.43 mmol) of NaBH_4_ was added to the solution, which was stirred at room temperature for 24 h. Then, a little portion of water was added and the product was extracted with dichloromethane. The organic layers were dried with anhydrous MgSO_4_ and the solvent was eliminated in vacuo to afford 0.0702 g (0.16 mmol, 75%) of *N*,*N′*-(1,4-phenylenebis(methylene))bis(3-(4-ethylpiperazin-1-yl)propan-1-amine) (**A**{*17*,*17*}) as a yellow oil. ^1^H-NMR (400 MHz, CDCl_3_) δ (ppm): 7.27 (s, 4H), 3.77 (s, 4H), 2.68 (t, J = 6.8 Hz, 4H), 2.64–2.25 (m, 22H), 2.07 (s, 4H), 1.72 (quint., *J* = 6.9 Hz, 4H), and 1.08 (t, *J* = 7.2 Hz, 6H). ^13^C-NMR (100.5 MHz, CDCl_3_) δ (ppm): 139.1, 128.3, 57.2, 53.8, 53.4, 53.0, 52.5, 48.3, 27.0, and 12.1. IR (KBr) ν_max_ (cm^−1^): 3421, 2943, 2807, 2399, 1545, 1470, 1403, 1339, 1311, 1271, 1166, 1015, 911, 836, 769, 657, and 560. HRMS (EI (70 eV)): calculated for C_26_H_48_N_6_ [M]^+^: 444.3940; found 444.3941.

***N*^1^,*N*^1^′-(1,4-phenylenebis(methylene))bis(*N*^3^-cyclohexyl-*N*^3^-methylpropane-1,3-diamine) A**{*18*,*18*}. The procedure was the same as that stated above for **11**{*17*,*17*} but was carried out by using 0.0770 g (0.45 mmol) of *N*^1^-cyclohexyl-*N*^1^-methylpropane-1,3-diamine (**9**{*18*}), 0.0303 g (0.23 mmol) of terephthalaldehyde (**12**), and 0.0173 g (0.46 mmol) of NaBH_4_ in 3 mL of anhydrous methanol to give 0.0677 g of *N*^1^,*N*^1^′-(1,4-phenylenebis(methylene))bis(*N*^3^-cyclohexyl-*N*^3^-methylpropane-1,3-diamine) (**A**{*18*,*18*}) (0.15 mmol, 68%) as a yellow oil. ^1^H-NMR (400 MHz, CDCl_3_) δ (ppm): 7.26 (s, 4H), 3.76 (s, 4H), 2.71–2.61 (m, 4H), 2.49 (t, J = 7.2 Hz, 4H), 2.40–2.28 (m, 2H), 2.23 (s, 6H), 1.82–1.56 (m, 14H), and 1.30–0.99 (m, 12H). ^13^C-NMR (100.5 MHz, CDCl_3_) δ (ppm): 139.0, 128.3, 62.8, 53.8, 52.1, 48.3, 37.8, 28.6, 27.9, 26.4, and 26.1. IR (KBr) ν_max_ (cm^−1^): 3423, 2929, 2853, 2800, 2406, 1540, 1450, 1391, 1262, 1222, 1199, 1162, 1125, 1052, 909, 890, 839, 768, 655, and 566. HRMS (EI (70 eV)): calculated for C_28_H_50_N_4_ [M]^+^: 442.4035; found 442.4002.

***N*^1^-cyclohexyl-*N*^3^-**(**4-**(((**3-**(**4-ethylpiperazin-1-yl**)**propyl**)**amino**)**methyl**)**benzyl**)**-*N*1-methylpropane-1,3-diamine A**{*17*,*18*}. For the first step, 0.13 mL (0.65 mmol) of 4-(diethoxymethyl)benzaldehyde (**16**), 0.1203 g (0.70 mmol) of 3-(4-ethylpiperazin-1-yl)propan-1-amine, and 2 mL of anhydrous methanol in the presence of anhydrous Na_2_SO_4_ were refluxed for 24 h under Ar atmosphere. Then, the solution was filtered, 0.0396 g (1.05 mmol) of NaBH_4_ was added, and the solution was stirred at room temperature for 12 h. The mixture was concentrated in vacuo and then a little portion of water was added. Next, the product was extracted with dichloromethane. The organic layers were dried with anhydrous MgSO_4_ and the solvent was removed under reduced pressure. The intermediate obtained was treated with 5 mL of 2 M HCl and the solution was stirred at room temperature for 2 h. The resulting mixture was neutralized with 1 M NaOH and it was extracted with dichloromethane. The combined organic layers were dried with anhydrous MgSO_4_ and the solvent was removed under reduced pressure. For the last step, the intermediate obtained (**17**{*17*}) was treated with the same procedure described in the first step but using 0.0547 g (0.32 mmol) of *N*^1^-cyclohexyl-*N*^1^-methylpropane-1,3-diamine (**9**{*18*}) as the amine to make the reaction. Finally, after the solvent removal under reduced pressure, 0.0849 g (0.19 mmol, 29%) of *N*^1^-cyclohexyl-*N*^3^-(4-(((3-(4-ethylpiperazin-1-yl)propyl)amino)methyl)benzyl)-*N*^1^-methylpropane-1,3-diamine (**A**{*17*,*18*}) was obtained. ^1^H-NMR (400 MHz, CDCl_3_) δ (ppm): 7.32 (s, 4H), 3.81 (d, J = 7.1 Hz, 4H), 2.75 (dt, J = 20.1, 6.5 Hz, 4H), 2.63 (t, J = 6.9 Hz, 2H), 2.56–2.35 (m, 13H), 2.31 (s, 3H), 1.88–1.69 (m, 8H), 1.64 (d, J = 12.7 Hz, 2H), 1.30–1.15 (m, 6H), and 1.13–1.03 (m, 5H). ^13^C-NMR (100.5 MHz, CDCl_3_) δ (ppm): 139.1, 138.7, 128.3, 128.2, 62.8, 57.1, 53.7, 53.4, 52.9, 52.4, 52.2, 48.3, 48.2, 37.7, 28.5, 27.7, 27.0, 26.4, 26.1, and 12.1. IR (ATR) ν_max_ (cm^−1^): 3271, 2926, 2851, 2805, 1448, 1347, 1308, 1164, 1116, 1116, 1016, and 803. HRMS (ESI-TOF): calculated for C_27_H_50_N_5_ [M + H]^+^: 444.4061; found 444.4061.

### 3.6. Cell Lines and Primary Cultures

DLBCL cell lines (HBL-1 and Karpas-422) used in this study were grown in Advanced-RMPI 1640 supplemented with 5% heat-inactivated FBS, 2 mmol/L of glutamine, and 50 µg/mL of penicillin–streptomycin (Thermo Fisher, Waltham, MA, USA). All cultures were routinely tested for Mycoplasma infection by PCR and the identity of all cell lines was verified by using an AmpFISTR identifier kit (Thermo Fisher).

Peripheral blood mononuclear cells (PBMCs) were obtained from buffy coats of healthy donors provided by the Catalan Blood and Tissue Biobank (agreement #190035). Once purified by standard Ficoll–Hypaque (GE Healthcare, Chalfont Saint Giles, UK) gradient centrifugation, the cells were cultured freshly as described above.

### 3.7. MTT Proliferation Assay

Antitumoral activity and selectivity were assessed using the MTT assay for cell viability and proliferation. Ten thousand DLBCL cells or 2 × 10^5^ PBMCs per well, respectively, were seeded in triplicate in a 96-well plate. The cells were treated with either vehicle control (DMSO) or the corresponding concentration (10, 30, 50, and 100 µM) of the indicated compound. The cells were then incubated for 24 h. After the incubation period, 10 µL/well of MTT solution (3-(4,5-Dimethylthiazol-2-yl)-2,5-diphenyltetrazolium bromide) (5 mg/mL in PBS) (Sigma-Aldrich, Saint-Quentin-Fallavier, France) was added, and the cells were further incubated for 4 additional hours. Subsequently, 100 µL of a 24:1 mixture of isopropanol and 1 M HCl was added to each well to solubilize the formazan crystals. The plate was incubated for 10 min at room temperature and protected from light. Cell viability was determined by measuring the absorbance at 560 nm using a 96-well plate reader. Background absorbance was subtracted using a medium-only control. The values are represented using untreated control cells as a reference. Half-maximal inhibitory (IC50) and lethal dose 50 (LD50) concentrations were defined as the doses of the different compounds needed to inhibit cell proliferation and cell viability, respectively, by 50%.

## 4. Conclusions

A virtual library of 225,750 tetraamines of general structure **8** was constructed by using five spacers (**A**–**E**) and 300 diamines **9** constructed from commercially available cyclic amines **10**. The combination of diversity selection, to reduce the molecular space without compromising its representativeness, and molecular docking to rank the selected molecules, has allowed us to find a potential candidate molecule with a high predicted affinity for CXCR4, showing the usefulness of molecular modeling and its decisive role in the identification of new hit compounds during the early stages of the drug discovery process.

Compound **A**{*17*,*18*} is our first example of a non-symmetrical compound including the p-phenylene moiety as the central core that presents activity in the micromolar range against DLBCL. It was synthesized together with its symmetrical structures **A**{*17*,*17*} and **A**{*18*,*18*}, and they were tested using the MTT cell viability and proliferation assay in DLBCL cell lines to assess their biological activity and in PBMCs to assess their cytotoxicity. The values obtained for **A**{*17*,*18*} (IC50 (Karpas422) = 39 µM, IC50 (HBL-1) = 45 µM) are better than **A**{*17*,*17*} but slightly lower than **A**{*18*,*18*}. However, it shows low toxicity, which makes it a promising starting molecule to design a potential drug candidate.

## Figures and Tables

**Table 1:** IC50 values obtained for the studied compounds against DLBCL cell lines and LD50 values measured on primary PBMC cultures.

	IC50 (µM)		LD50 (µM)
	Karpas422	HBL-1	PBMCs
**A**{*17*,*17*}	>50	>50	>50
**A**{*17*,*18*}	39	45	>50
**A**{*18*,*18*}	36	20	23

## Data Availability

Data is contained within the article or Supplementary Material.

## Supplementary Materials

The following supporting information can be downloaded at https://www.mdpi.com/article/10.3390/ijms25179446/s1.
