# Supplementary material for: Applying Molecular Modeling to the Design of Innovative, Non-Symmetrical CXCR4 Inhibitors with Potent Anticancer Activity
Source: Int J Mol Sci. 2024 Aug 30;25(17):9446. doi: 10.3390/ijms25179446 (PMC11394923; doi:10.3390/ijms25179446)
Supplement: Supplementary file 1 [file ijms-25-09446-s001.zip › ijms-3166217-supplementary.pdf]

# Applying Molecular Modeling to the Design of Innovative, Non-Symmetrical CXCR4 Inhibitors with Potent Anticancer Activity

*Miquel Martínez-Asensio[1], Lluís Sàrrias[1], Gema Gorjón-de-Pablo[1,2], Miranda Fernández-Serrano[2], Judith Camaló-Vila[1], Albert Gibert[1], Raimon Puig de la Bellacasa[1], Jordi Teixidó[1], Gaël Roué[2], José I. Borrell[1], Roger Estrada-Tejedor [1]**

[1] Grup de Química Farmacèutica, IQS School of Engineering, Universitat Ramon Llull, Via Augusta 390, E-08017 Barcelona, Spain

[2] Lymphoma Translational Group, Josep Carreras Leukaemia Research Institute, 08916 Badalona, Spain

Corresponding author: roger.estrada@iqs.url.edu

## *Supporting Information*

Table S1. List of molecular descriptors ..................................................................... pg. 2

Table S2. List of 10 compounds with better docking score values ............................ pg. 3

Figure S1. Result of the validation docking .............................................................. pg. 4

Figure S2. Root mean square deviation (RMSD) obtained in molecular dynamics simulations.................................................................................................. pg. 5

Appendix S1. NMR and IR Spectra ............................................................................ pg. 6

**Table S1. List of molecular descriptors used in library selection**

| | | | | | |
|---|---|---|---|---|---|
| a_acc | b_1rotN | diameter | lip_don | PEOE_VSA_NEG | RPC+ |
| a_acid | b_1rotR | FCharge | lip_druglike | PEOE_VSA_PNEG | rsynth |
| a_aro | b_ar | GCUT_PEOE [0-3] | lip_violation | PEOE_VSA_POL | SlogP |
| a_base | b_count | GCUT_SLOGP [0-3] | logP(o/w) | PEOE_VSA_POS | SlogP_VSA [0,9] |
| a_count | b_double | GCUT_SMR [0-3] | logS | PEOE_VSA_PPOS | SMR |
| a_don | b_heavy | h_ema | mr | petitjean | SMR_VSA [0,7] |
| a_donacc | b_max1len | h_emd | mutagenic | petitjeanSC | TPSA |
| a_heavy | b_rotN | h_emd_C | nmol | Q_PC- | VAdjEq |
| a_hyd | b_rotR | h_log_dbo | opr_brigid | Q_PC+ | VAdjMa |
| a_IC | b_single | h_log_pbo | opr_leadlike | Q_RPC- | VDistEq |
| a_ICM | b_triple | h_logD | opr_nring | Q_RPC+ | VDistMa |
| a_nB | balabanJ | h_logP | opr_nrot | Q_VSA_FHYD | vdw_area |
| a_nBr | BCUT_PEOE [0-3] | h_logS | opr_violation | Q_VSA_FNEG | vdw_vol |
| a_nC | BCUT_SLOGP [0-3] | h_mr | PC- | Q_VSA_FPNEG | vsa_acc |
| a_nCl | BCUT_SMR [0-3] | h_pavgQ | PC+ | Q_VSA_FPOL | vsa_acid |
| a_nF | bpol | h_pKa | PEOE_PC- | Q_VSA_FPOS | vsa_base |
| a_nH | chi0 | h_pKb | PEOE_PC+ | Q_VSA_FPPOS | vsa_don |
| a_nI | chi0_C | h_pstates | PEOE_RPC- | Q_VSA_HYD | vsa_hyd |
| a_nN | chi0v | h_pstrain | PEOE_RPC+ | Q_VSA_NEG | vsa_other |
| a_nO | chi0v_C | Kier1 | PEOE_VSA [-6,+6] | Q_VSA_PNEG | vsa_pol |
| a_nP | chi1 | Kier2 | PEOE_VSA_FHYD | Q_VSA_POL | Weight |
| a_nS | chi1_C | Kier3 | PEOE_VSA_FNEG | Q_VSA_POS | weinerPath |
| apol | chi1v | KierA1 | PEOE_VSA_FPNEG | Q_VSA_PPOS | weinerPol |
| ast_fraglike | chi1v_C | KierA2 | PEOE_VSA_FPOL | radius | zagreb |
| ast_fraglike_ext | chiral | KierA3 | PEOE_VSA_FPOS | reactive | |
| ast_violation | chiral_u | KierFlex | PEOE_VSA_FPPOS | rings | |
| ast_violation_ext | density | lip_acc | PEOE_VSA_HYD | RPC- | |

**Table S2. List of 10 compounds with better docking score values.**

| Compound | ID | S |
|---|---|---|
| 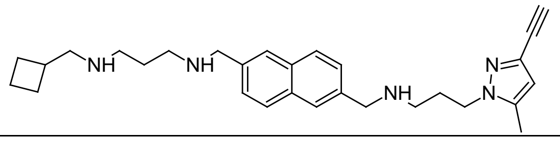 | 61 | -6.3 |
| 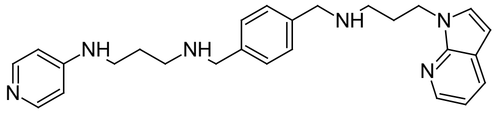 | 23 | -6.3 |
| 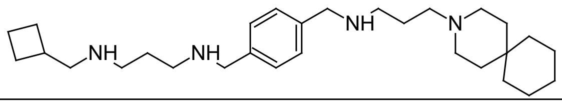 | 19 | -6.1 |
| 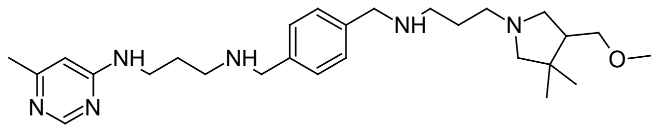 | 36 | -6.1 |
| 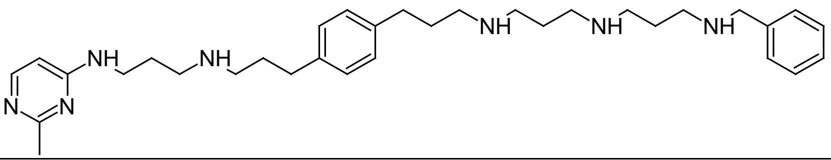 | 122 | -6.0 |
| 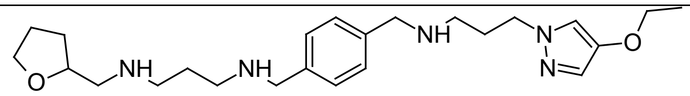 | 32 | -6.0 |
| 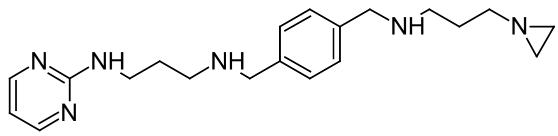 | 3 | -6.0 |
| 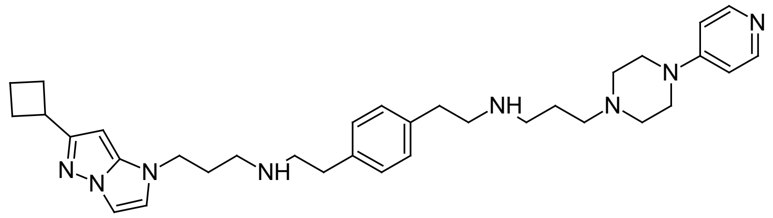 | 102 | -5.9 |
| 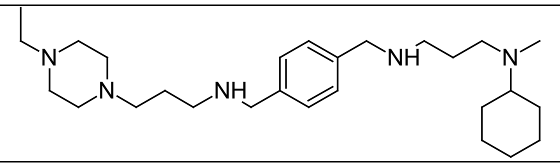 | 11 | -5.7 |
| 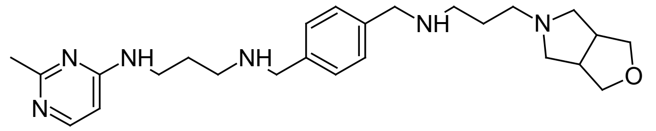 | 37 | -5.6 |

**Figure S1. Result of the validation docking.** The IT1t structure obtained from the validation docking (green) was superposed with the conformation reported in the PDB (3ODU, red), showing an RMSD of 0.96.

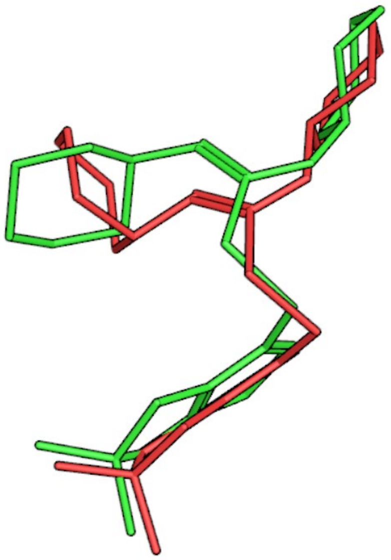

**Figure S2. Root mean square deviation (RMSD) obtained in molecular dynamics simulations.**

RMSD obtained for the ligands under study during the simulation. The RMSD (in Å) was calculated considering all non-hydrogen atoms in the ligand molecule.

Ligands converge, in all simulations, to a metastable disposition with an RMSD < 1 Å difference.

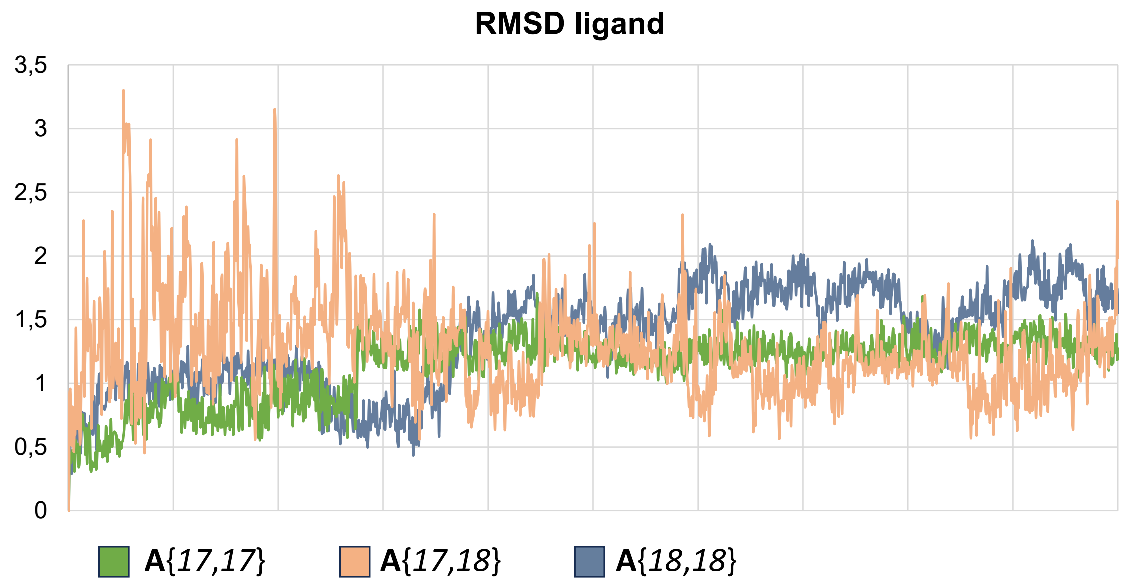

## Appendix S1. NMR and IR Spectra

*N*,*N'*-(1,4-phenylenebis(methylene))bis(3-(4-ethylpiperazin-1-yl)propan-1-amine) (**A**{*17*,*17*})

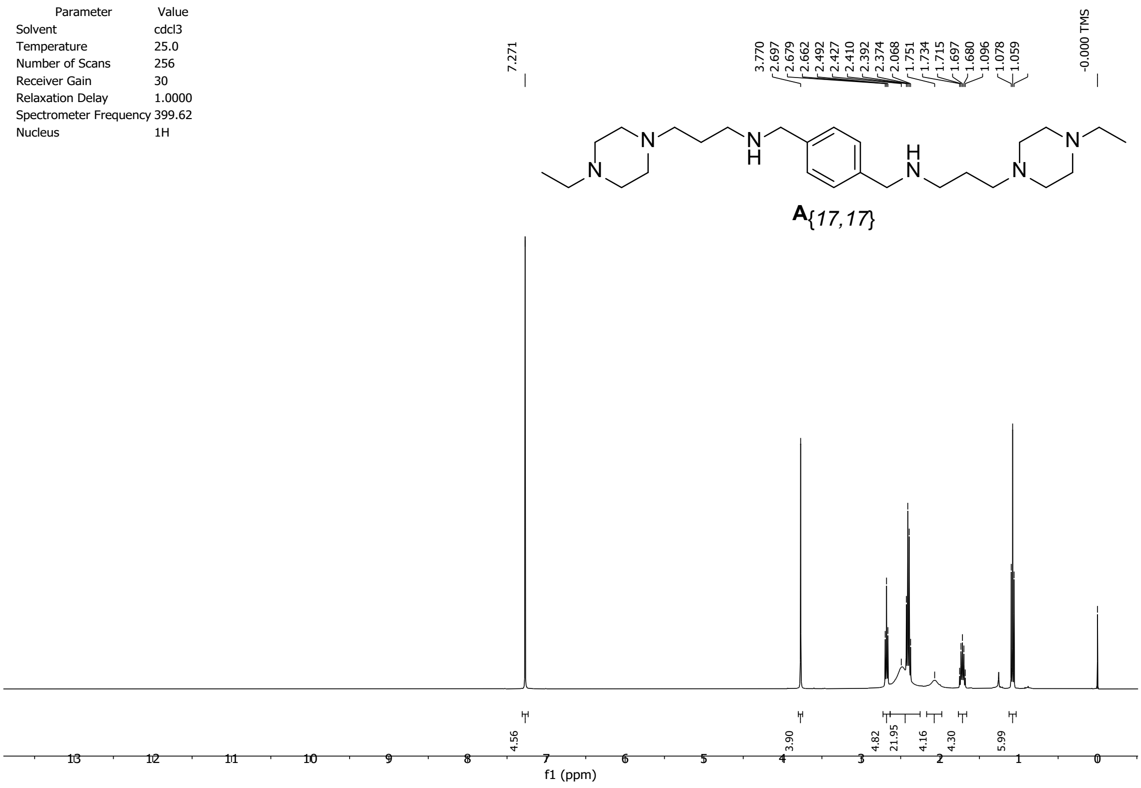


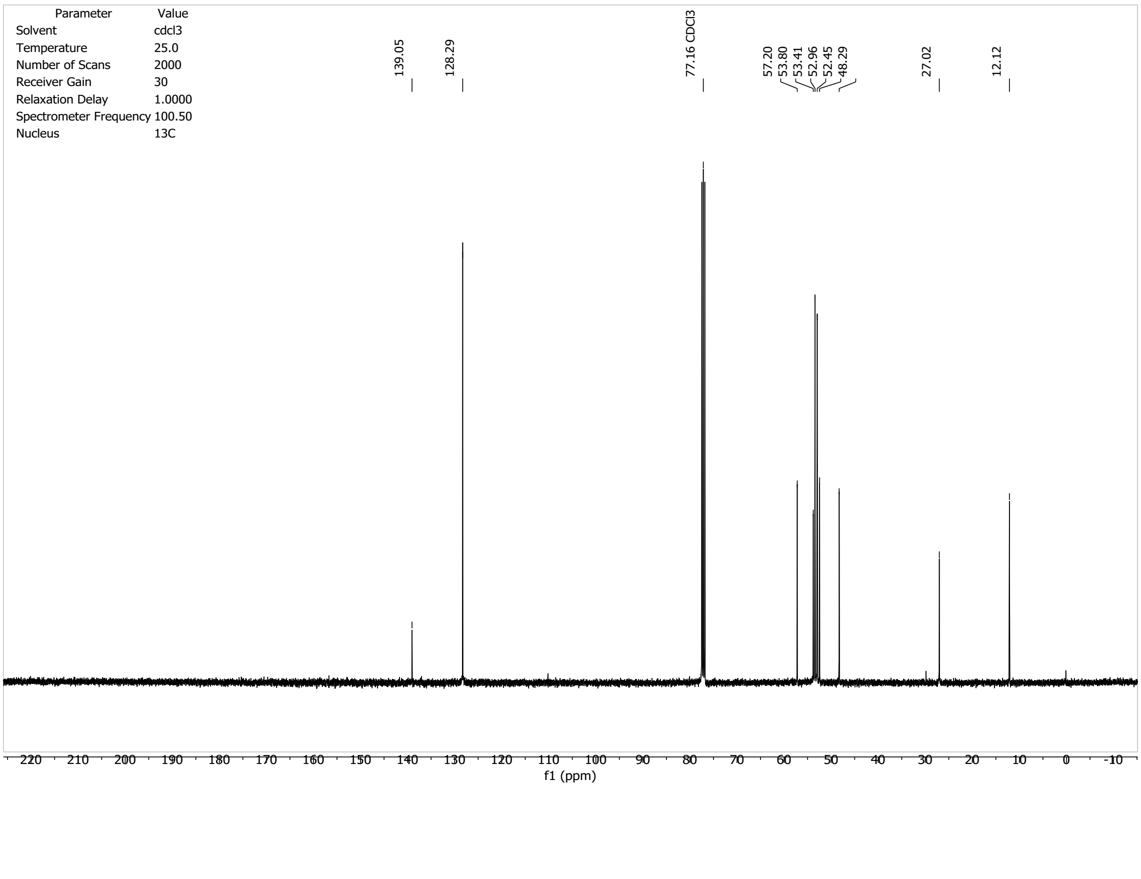

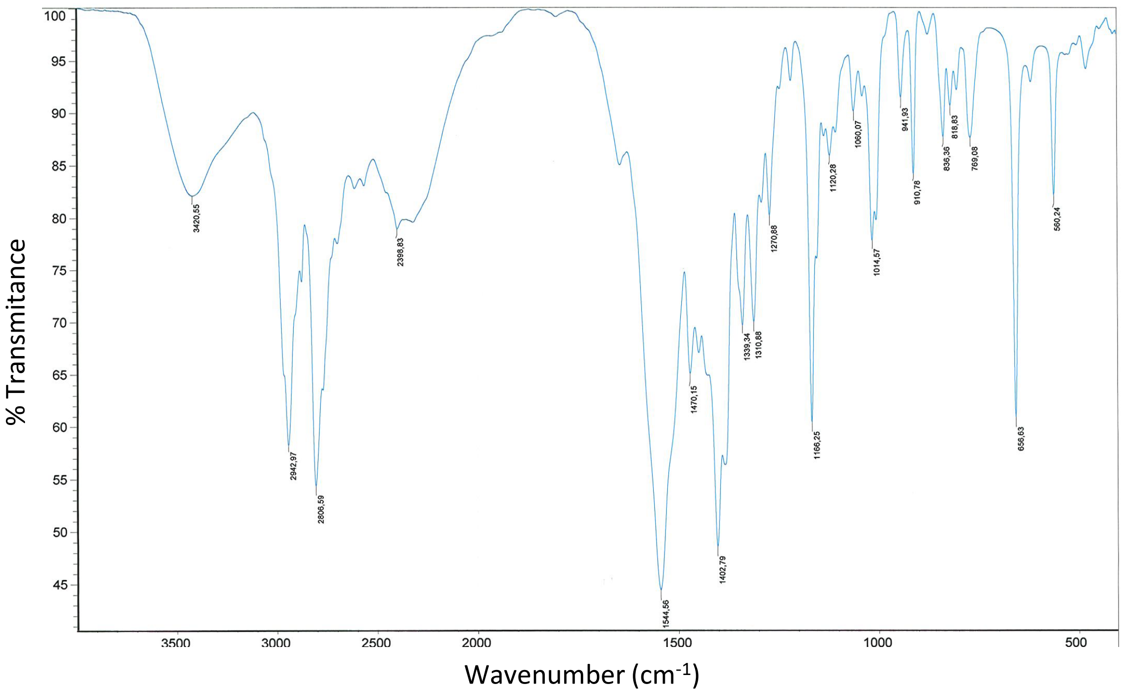
% Transmitance
100
95
90
85
80
75
70
65
60
55
50
45
3500
3000
2500
2000
1500
1000
500
Wavenumber (cm-1)
3420,55
2942,97
2806,59
2398,83
1544,56
1470,15
1402,79
1339,34
1310,88
1270,88
1166,25
1120,28
1060,07
1014,57
941,93
910,78
836,36
818,83
769,08
656,63
560,24

$N^1$,$N^{1'}$-(1,4-phenylenebis(methylene))bis($N^3$-cyclohexyl-$N^3$-methylpropane-1,3-diamine) (**A**{*18*,*18*})

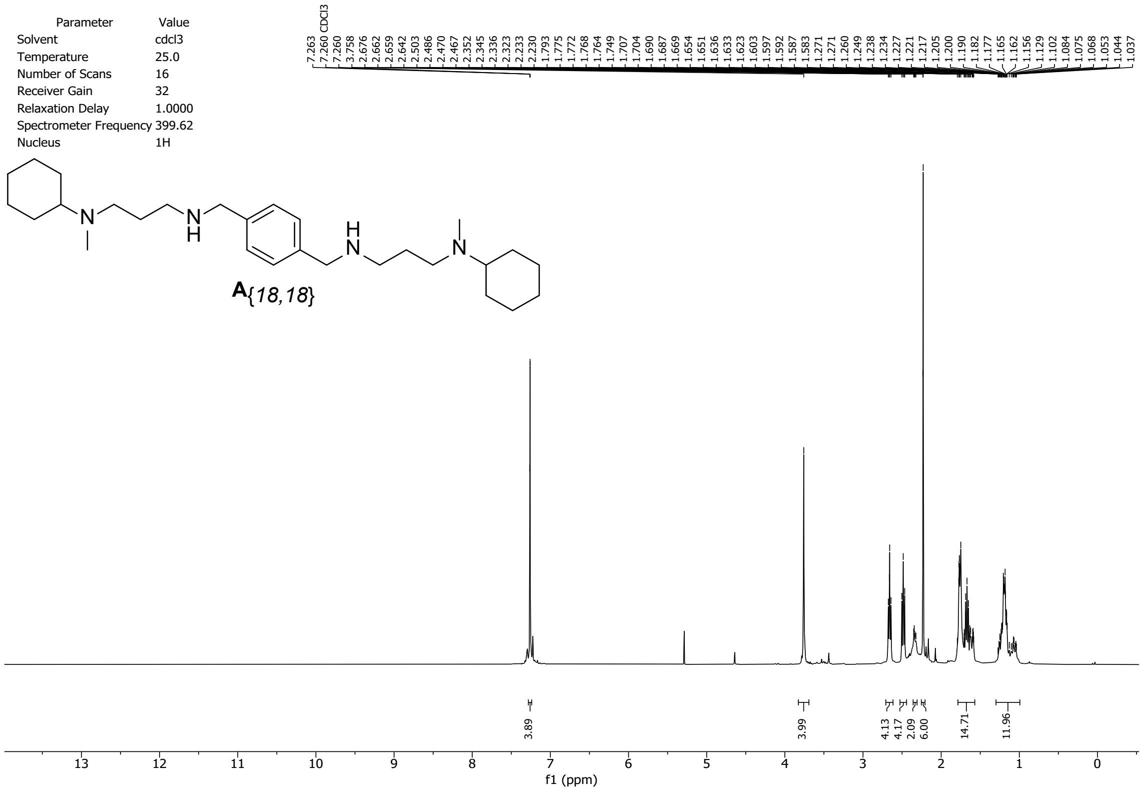


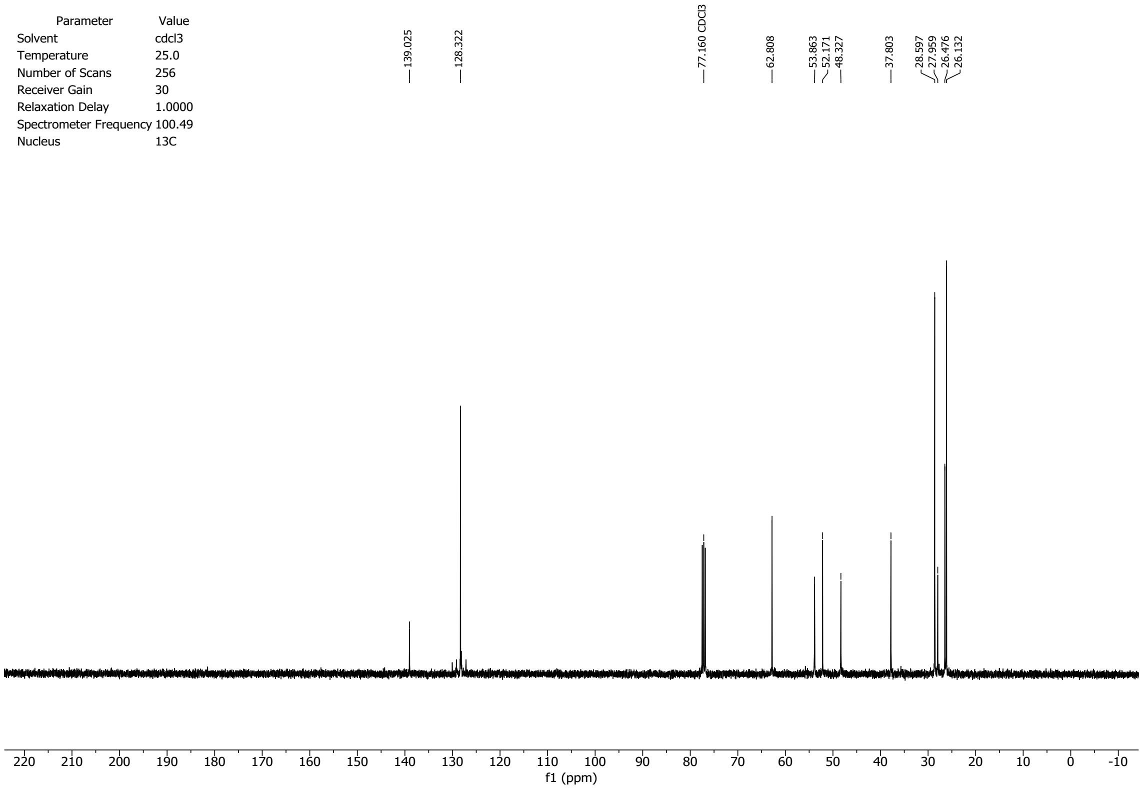

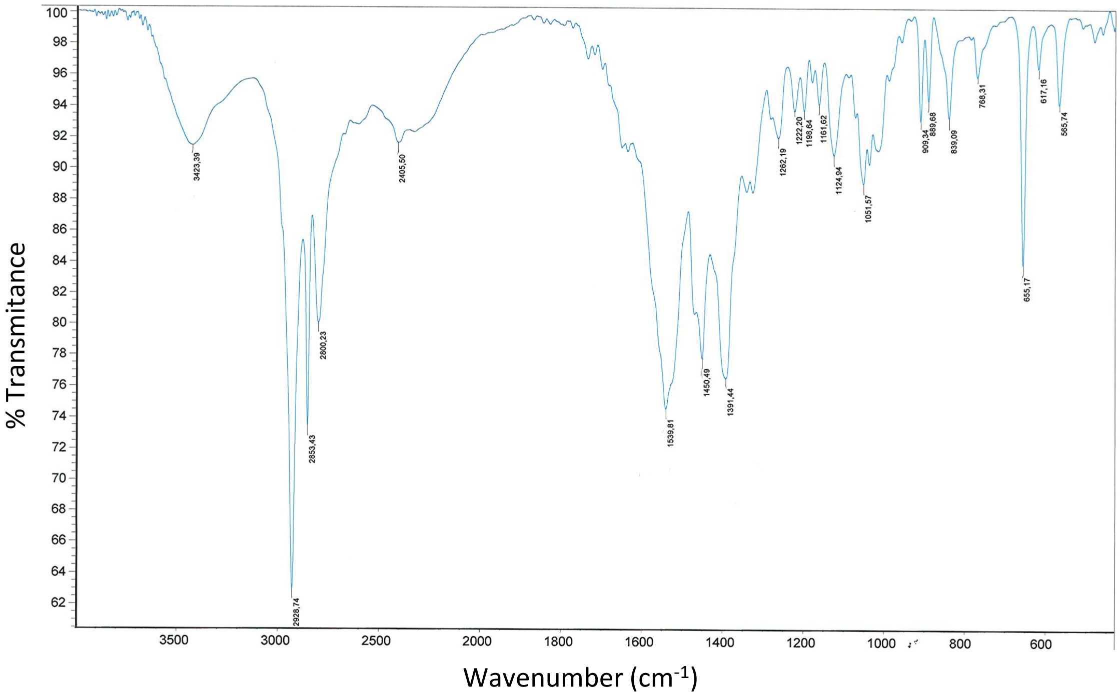
% Transmitance
Wavenumber (cm-1)
100
98
96
94
92
90
88
86
84
82
80
78
76
74
72
70
68
66
64
62
3500
3000
2500
2000
1800
1600
1400
1200
1000
800
600
3423,39
2928,74
2853,43
2800,23
2405,50
1539,81
1450,49
1391,44
1262,19
1222,20
1198,64
1161,62
1124,94
1051,57
909,34
889,68
839,09
768,31
655,17
617,16
565,74

*N*[1]-cyclohexyl-*N*[3]-(4-(((3-(4-ethylpiperazin-1-yl)propyl)amino)methyl)benzyl)-*N*[1]-methylpropane-1,3-diamine (**A**{*17*,*18*})

| Parameter | Value |
|---|---|
| Solvent | cdcl3 |
| Temperature | 25.0 |
| Number of Scans | 32 |
| Receiver Gain | 50 |
| Relaxation Delay | 1.0000 |
| Spectrometer Frequency | 399.62 |
| Nucleus | 1H |

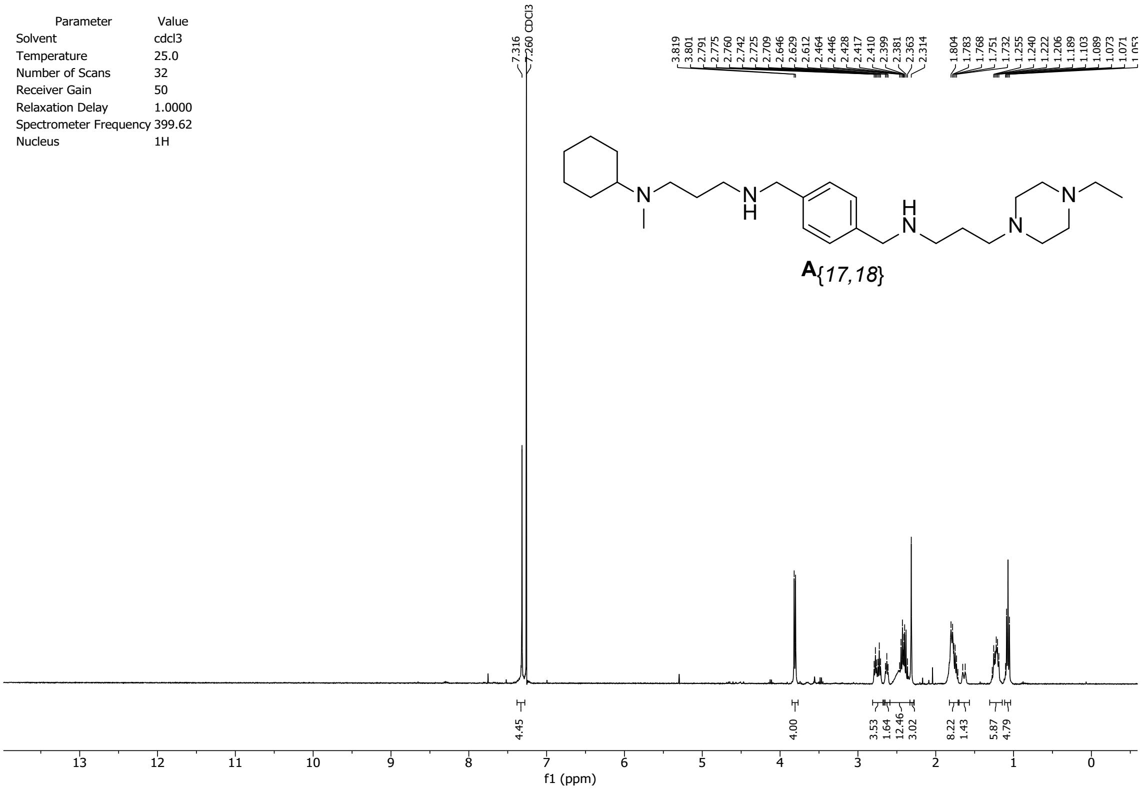


| Parameter | Value |
|---|---|
| Solvent | cdcl3 |
| Temperature | 25.0 |
| Number of Scans | 2000 |
| Receiver Gain | 30 |
| Relaxation Delay | 1.0000 |
| Spectrometer Frequency | 100.49 |
| Nucleus | 13C |

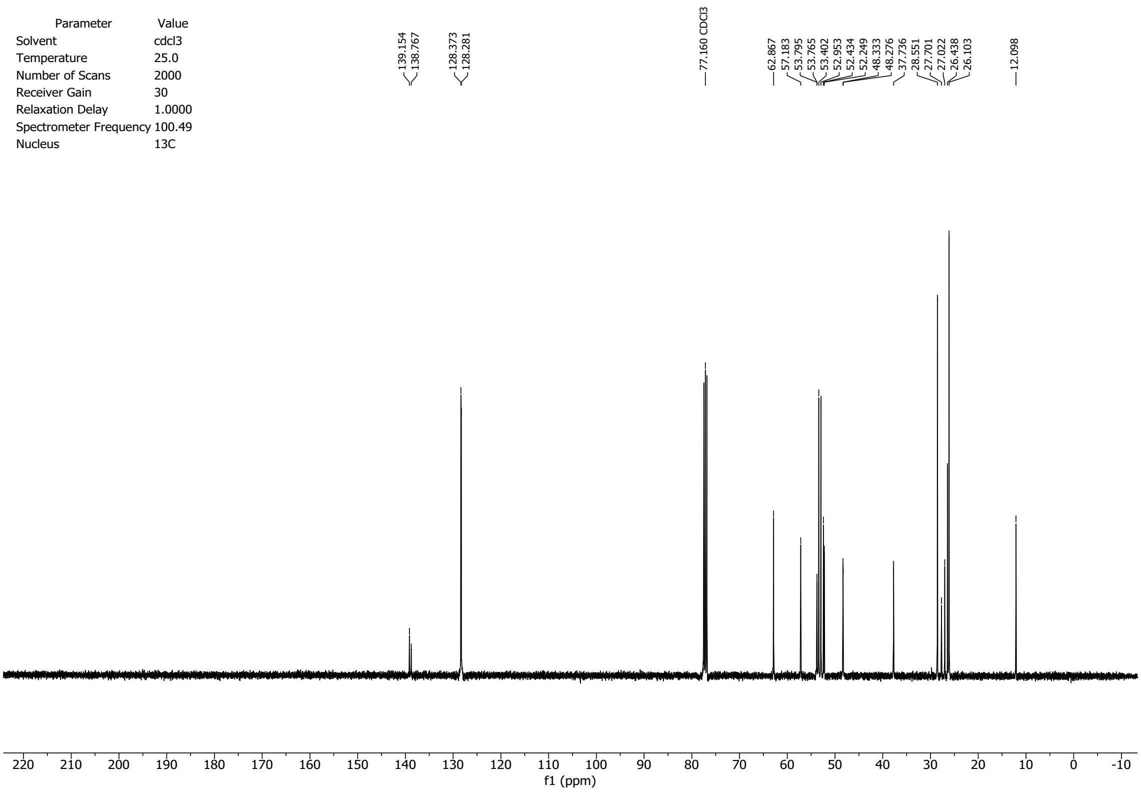

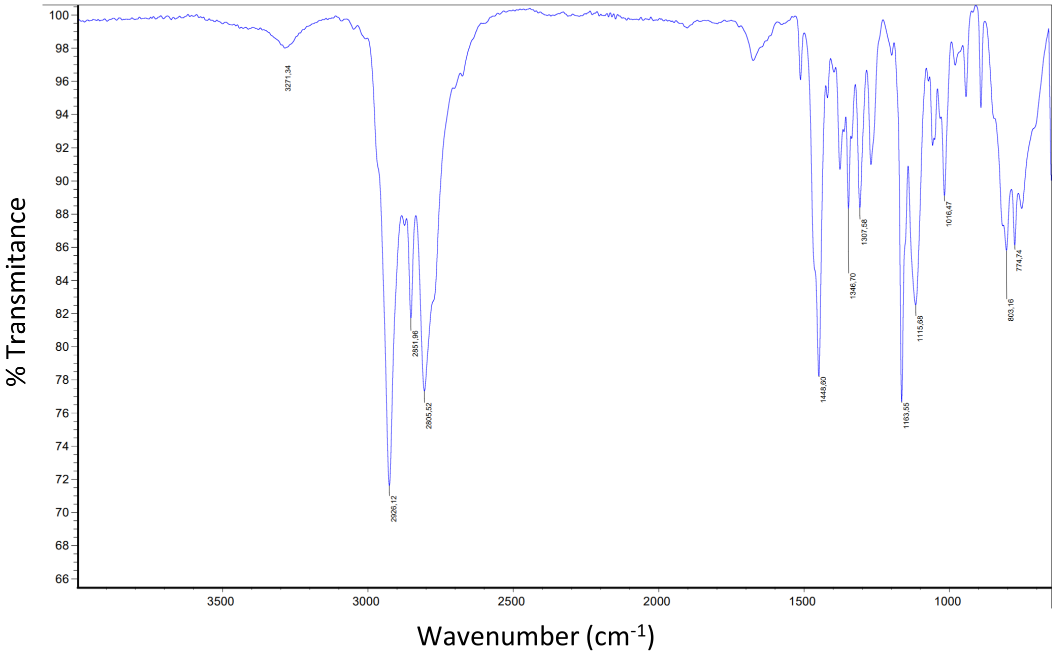
% Transmitance
Wavenumber (cm-1)
100
98
96
94
92
90
88
86
84
82
80
78
76
74
72
70
68
66
3500
3000
2500
2000
1500
1000
3271,34
2926,12
2851,96
2805,52
1448,60
1346,70
1307,58
1163,55
1115,68
1016,47
803,16
774,74
